# Evaluation of antibiotic escalation in response to nurse-driven inpatient sepsis screen

**DOI:** 10.1017/ash.2021.232

**Published:** 2021-12-03

**Authors:** Daisuke Furukawa, Thomas D. Dieringer, Mitchell D. Wong, Julia T. Tong, Isa A. Cader, Lauren E. Wisk, Maria A. Han, Summer M. Gupta, Russell B. Kerbel, Daniel Z. Uslan, Christopher J. Graber

**Affiliations:** 1 Division of Infectious Disease, Department of Medicine, University of California–Los Angeles, California; 2 Division of General Internal Medicine, Department of Medicine, University of California, Los Angeles, California; 3 David Geffen School of Medicine, University of California–Los Angeles, California; 4 Quality Management Services, UCLA Health, Los Angeles, California; 5 Infectious Diseases Section, VA Greater Los Angeles Healthcare System, Los Angeles, California

## Abstract

**Objective:**

To determine the frequency and predictors of antibiotic escalation in response to the inpatient sepsis screen at our institution.

**Design:**

Retrospective cohort study.

**Setting:**

Two affiliated academic medical centers in Los Angeles, California.

**Patients:**

Hospitalized patients aged 18 years and older who had their first positive sepsis screen between January 1, 2019, and December 31, 2019, on acute-care wards.

**Methods:**

We described the rate and etiology of antibiotic escalation, and we conducted multivariable regression analyses of predictors of antibiotic escalation.

**Results:**

Of the 576 cases with a positive sepsis screen, antibiotic escalation occurred in 131 cases (22.7%). New infection was the most documented etiology of escalation, with 76 cases (13.2%), followed by known pre-existing infection, with 26 cases (4.5%). Antibiotics were continued past 3 days in 17 cases (3.0%) in which new or existing infection was not apparent. Abnormal temperature (adjusted odds ratio [aOR], 3.00; 95% confidence interval [CI], 1.91–4.70) and abnormal lactate (aOR, 2.04; 95% CI, 1.28–3.27) were significant predictors of antibiotic escalation. The patient already being on antibiotics (aOR, 0.54; 95% CI, 0.34–0.89) and the positive screen occurred during a nursing shift change (aOR, 0.36; 95% CI, 0.22–0.57) were negative predictors. Pneumonia was the most documented new infection, but only 19 (50%) of 38 pneumonia cases met full clinical diagnostic criteria.

**Conclusions:**

Inpatient sepsis screening led to a new infectious diagnosis in 13.2% of all positive sepsis screens, and the risk of prolonged antibiotic exposure without a clear infectious source was low. Pneumonia diagnostics and lactate testing are potential targets for future stewardship efforts.

Antibiotic overuse is a national healthcare problem. Studies have demonstrated that up to 32%–60% of hospitalized patients are on antibiotics at a given time, of which about one-third are considered inappropriate.^1–5^ Overuse contributes to several adverse outcomes including increases in *Clostridioides difficile* infections, which are associated with significant mortality and excess medical costs,^6^ multidrug-resistant organisms (eg, carbapenem-resistant *Enterobacterales*), allergic reactions, and other side effects such as kidney injury.^6,7^ Judicious antibiotic use is thus essential in reducing development of resistant organisms and optimizing patient outcomes.

On the other hand, sepsis is a common cause of in-hospital mortality and has been identified as a national healthcare priority. The Surviving Sepsis Campaign guidelines and the Center for Medicare and Medicaid Service (CMS) Core Measure SEP-1 mandate have emphasized timely administration of antibiotics to patients suspected of having sepsis.^8,9^ Despite these recommendations, evidence supporting aggressive early treatment for patients with sepsis without septic shock is mixed.^10,11^ Furthermore, the Infectious Diseases Society of America (IDSA) has recently recommended removing sepsis without shock from the SEP-1 mandate to reduce the risk of unintended overuse of antibiotics.^12^ The priorities of antibiotic stewardship and sepsis management may be conflicting, and studies evaluating unintended consequences of sepsis treatment guidelines and national mandates are of utmost importance.

Different sepsis screening tools have been implemented at various institutions, ranging from nurse-driven manual screens to completely automated screens. Although studies have shown that these screening tools generally improve process measures for sepsis care (eg, timely antibiotic administration), mortality benefits have not been clearly demonstrated.^13^ Furthermore, these screens have highly variable sensitivity and specificity for actual sepsis, often with low positive predictive value (ie, 10% in one study).^13,14^ This situation has raised the question of whether screening for sepsis and promoting overrecognition and treatment can contribute to unnecessary antibiotic use. In this study, we characterized the rate and etiology of antibiotic escalation in response to the inpatient sepsis screen at our institution.

## Methods

### Nurse-driven sepsis screen

At our institution, the sepsis screen is a nurse-driven screening tool embedded in the electronic medical record that the oncoming and off-going nurses jointly review for each patient at change of shift (7 a.m. and 7 p.m.), when patients are admitted or transferred to a new unit, and whenever severe sepsis is suspected by the nurse. All nurses underwent mandatory sepsis education, and specific training on the sepsis screen was also provided for newly hired nurses. The complete list of questions and criteria included in the sepsis screen can be found in the Supplementary Material (Supplementary Table 1). The screen is considered positive when an infection is suspected by the nurse, at least 2 systemic inflammatory response syndrome (SIRS) criteria are met anytime in the preceding 12-hour window, and there is evidence of at least 1 organ dysfunction not related to chronic conditions or medications. When the screen is positive, the nurse is responsible for notifying the responsible physician and initiating sepsis bundle elements including drawing lactate levels and blood cultures. However, nurses are not authorized to initiate antibiotics unless an order is placed by a physician.

### Chart review

We retrospectively reviewed the electronic medical records of patients who were admitted to 1 of 2 medical centers, a 500-bed, academic quaternary referral center, and an affiliated 250-bed, academic medical center in Los Angeles from January 1, 2019, to December 31, 2019. These 2 medical centers share the same electronic medical record and share trainees from the same residency and fellowship programs. The inclusion criteria for the study included all patients aged 18 years and older admitted during the evaluation period who also had a positive inpatient sepsis screen anytime during the hospitalization. Only the first positive sepsis screen after admission for each hospitalization was considered for analysis; thus, subsequent positive screens were excluded. Additionally, analysis was limited to positive screens that happened on the inpatient acute-care wards; thus, positive screens in the emergency department or in the intensive care unit were excluded. Lastly, we also excluded patients who were neutropenic or admitted to the bone marrow transplant unit because antibiotics are often protocolized in these clinical situations.

Our primary outcome was rate of antibiotic escalation, and secondary outcomes were etiology and predictors of antibiotic escalation. We abstracted data including patient demographics, vital signs, and laboratory values by manual chart review. For each vital sign of interest, the most abnormal value recorded within 12 hours prior to and 3 hours after the sepsis screen was abstracted. This 15-hour window was selected to incorporate the 12-hour review period integrated in the sepsis screen and to include vital signs recorded while the primary provider was evaluating the patient in response to the positive screen. Similarly, for laboratory tests of interest, we abstracted the most abnormal value recorded within 24 hours prior to and 3 hours after the sepsis screen. A 24-hour review window was used for laboratories because laboratory values were often missing for cases when sepsis screen happened at night if a 12-hour window was used. Antibiotic escalation was defined as initiation of antibiotics, addition of antibiotics, or switching antibiotics to another agent with equal or increased spectrum of activity within 3 hours of positive sepsis screen. Antibiotic ranks based on spectrum of activity can be found in the Supplementary Material (Supplementary Table 2a and b). We additionally reviewed medical charts to assess for presence of and reason for antibiotic escalation. When antibiotics were escalated for possible pneumonia, the clinical criteria met for diagnosis of pneumonia were evaluated using the definition outlined in the 2016 Infectious Diseases Society of America (IDSA) and American Thoracic Society (ATS) hospital-acquired and ventilator-acquired pneumonia guidleines.^15^ Patients were considered to meet diagnostic criteria for pneumonia if they had evidence of progressive infiltrate on imaging and met 2 of 3 clinical criteria: white blood cell count (WBC) ≥12 or <4, temperature ≥38°C or <36°C, and/or purulent sputum production. The Institutional Review Board of the University of California–Los Angeles approved the study.

### Statistical analysis

Results were first analyzed descriptively. We used the χ^2^ test for categorical variables, the Student *t* test for normally distributed continuous variables, and the Wilcoxon rank-sum test for continuous variables that were not normally distributed. Multivariable logistic regression analysis was used to evaluate predictors of antibiotic escalation. Components of past and current definitions of sepsis, severe sepsis, and septic shock were retained in the model.^16,17^ Additionally, timing of the sepsis screen relative to provider shift, patient immunosuppression, provider medical specialty, and patient already being on antibiotics were also included because these factors were noted to influence antibiotic prescribing behaviors in past studies or the authors hypothesized them to influence antibiotic prescribing behavior.^18,19^ We used STATA version 16 software (STATA Corp, College Station, TX) for all analyses.

## Results

**Fig. 1.**
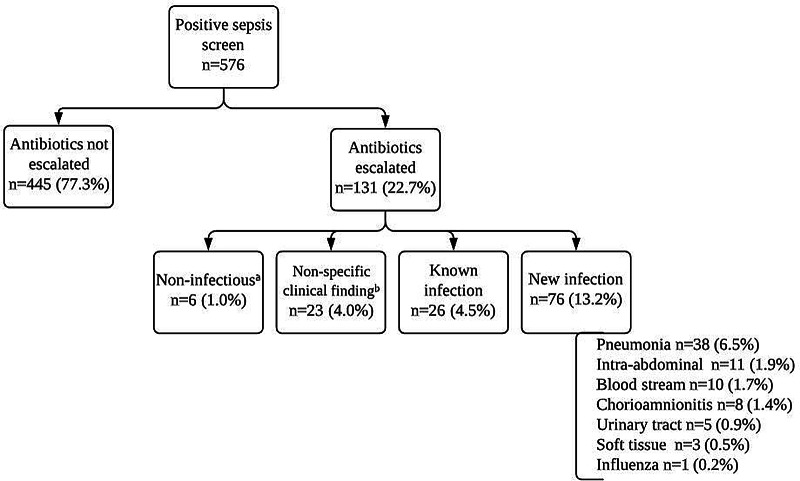
Summary of cases with positive sepsis screen with etiologies of antibiotic escalation. ^a^Pneumonitis, atrial fibrillation, pancreatitis, COPD exacerbation, and bronchiectasis flare. ^b^Hypotension, leukocytosis, hypoxia, hypercapnia, fever, and tachycardia.

Figure 1 provides a summary of the cases of positive sepsis screen included in our analysis. In total, 576 consecutive patients were included based on our inclusion and exclusion criteria. Among them, antibiotic escalation was seen in 131 (22.7%) cases. New infection was the most documented reason for antibiotic escalation (n = 76; 58.0% of escalation cases), accounting for 13.2% of all positive sepsis screens. Pneumonia accounted for half (n = 38) of the presumed new infections. In 26 cases (4.5% of all positive screens), antibiotics were escalated in the setting of a known infection but were subsequently deescalated within 3 days after a negative work-up for a new infection. Of the remaining 29 cases in which antibiotics were escalated, 6 had noninfectious conditions that led to escalation (ie, pneumonitis, atrial fibrillation, pancreatitis, exacerbation of chronic obstructive pulmonary disease) and 23 had nonspecific clinical findings that alone were not suggestive of true infection (eg, hypotension, leukocytosis, hypoxia, hypercapnia, fever, or tachycardia). Antibiotics were continued past 3 days in 17 of these 29 cases (3.0% of positive sepsis screens overall) despite not having a documented infectious source.

Patients who had antibiotics escalated were less likely to have experienced the sepsis screen at change of shift compared to patients who did not have antibiotics escalated. (22.9% vs 49.4%; *P* < .001) (Table 1). The escalated antibiotic group was also less likely to already be on antibiotics at time of sepsis screen (64.9% vs 73.7%; *P* = .049) and more likely to have an abnormal temperature (67.9% vs 42.0%; *P* < .001), abnormal lactate (33.6% vs 18.7%; *P* < .001), and higher SIRS score (3.7 vs 2.5; *P* < .001) compared to those not escalated. The escalated and nonescalated antibiotics groups were similar in other clinical characteristics such as comorbid conditions, abnormal white cell count, sequential organ failure assessment (SOFA) score, and code status.

**Table 1.** Characteristics of Patients Grouped by Antibiotics Escalation

Patient Characteristics	No Escalation No. (%)	Escalation No. (%)	*P* Value
**Total**	445	131	
**Demographics**			
Age, mean y ±SD y	63.8 ±19.0	63.5 ±18.0	.886
Male	223 (50.1)	62 (47.3)	.575
**Hospital/Setting characteristics**			
Medicine service	305 (68.5)	88 (67.2)	.768
Postoperative	162 (36.4)	52 (39.7)	.493
Shift change^a^	220 (49.4)	30 (22.9)	<.001
Night shift	207 (46.5)	54 (41.2)	.285
**Comorbid conditions**			
Diabetes mellitus	99 (22.3)	36 (27.5)	.214
Chronic kidney disease	48 (10.8)	15 (11.5)	.837
Chronic heart disease	120 (27.0)	39 (29.8)	.528
Cirrhosis	31 (6.97)	15 (11.5)	.096
Chronic lung disease	78 (17.5)	30 (22.9)	.166
Immunosuppressed	123 (27.6)	34 (26.0)	.703
History of multidrug-resistant organisms^b^	58 (13.1)	19 (14.5)	.670
**Clinical variables**			
On antibiotics	328 (73.7)	85 (64.9)	.049
Antibiotics escalated 24 h prior	122 (30.4)	40 (23.0)	.086
Total SIRS, mean ±SD	2.5 ±0.7	3.7 ±0.8	.003
SOFA score, mean ±SD	6.4 ±1.2	6.6 ±1.3	.095
Abnormal temperature: <36 or >38°C	187 (42.0)	89 (67.9)	<.001
Abnormal heart rate: >90 beats/min	390 (87.6)	114 (87.0)	.851
Abnormal respiratory rate:>20 respiration/min	289 (64.9)	90 (68.7)	.425
Abnormal WBC: >12,000/uL or < 4,000/uL or >10% bands	251 (57.2)	66 (51.2)	.227
Abnormal blood pressure: <90 systolic blood pressure or <65 mean arterial pressure	118 (26.5)	36 (27.7)	.790
Abnormal lactate: >18 mg/dL	83 (18.7)	44 (33.6)	<.001
Glasgow coma scale, mean ±SD	14.2 ±1.8	13.9 ±2.2	.167
Do not resuscitate code status	69 (15.5)	26 (20.0)	.230

Note. SD, standard deviation; SIRS, systemic inflammatory response syndrome; SOFA, sequential organ-failure assessment.

a Shift change refers to sepsis screens that happened routinely at nurse’s change of shift as opposed to the screen triggering mid-shift when the nurse suspected sepsis.

b Multidrug-resistant organism defined as carbapenem-resistant gram-negative rod, extended-spectrum β-lactamase–producing *Enterobacterales*, vancomycin-resistant *enterococcus,* methicillin resistant *Staphylococcus aureus*.

On multivariable regression analysis, abnormal temperature (adjusted odds ratio [aOR], 3.00; 95% confidence interval [CI], 1.91–4.70) and abnormal lactate (aOR, 2.04; 95% CI, 1.28–3.27) were significant predictors of antibiotic escalation (Table 2). The patient being on antibiotics (aOR, 0.54; 95% CI, 0.34–0.89) and the positive screen occurring during a nursing shift change (aOR, 0.36; 95% CI, 0.22–0.57) were negative predictors of antibiotic escalation.

**Table 2.** Multivariable Logistic Regression Analysis for Predictors of Antibiotic Escalation

Variable	Adjusted OR	95% CI	*P* Value
Medicine vs surgical service	1.27	0.80–2.02	0.316
Immunosuppressed (yes vs no)	0.98	0.61–1.60	0.947
On antibiotics (yes vs no)	0.54	0.34–0.89	0.009
Abnormal temperature (yes vs no)	3.00	1.91–4.70	<0.001
Abnormal heart rate (yes vs no)	1.05	0.56–1.97	0.884
Abnormal respiratory rate (yes vs no)	1.45	0.92–2.30	0.110
Abnormal WBC (yes vs no)	0.81	0.53–1.26	0.355
Abnormal blood pressure (yes vs no)	1.03	0.64–1.65	0.902
Abnormal lactate (yes vs no)	2.04	1.28–3.27	0.003
Shift change^a^ (yes vs no)	0.36	0.22–0.57	<0.001
Night vs day	0.69	0.45–1.07	0.099

Note. OR, odds ratio; CI, confidence interval.

a Shift change refers to sepsis screens that happened routinely at nurse’s change of shift as opposed to the screen triggering midshift when the nurse suspected sepsis.

Pneumonia was the most common infectious diagnosis made after a positive sepsis screen, accounting for 38 (50.0%) of 76 new infections (Fig. 1). Of the 38 cases of pneumonia, only 19 met full diagnostic criteria for pneumonia, and 4 did not have radiologic evidence of pneumonia (Table 3).

**Table 3.** Number of Clinical Criteria Met for Presumed Pneumonia Diagnoses

Clinical Criteria^a^ for Pneumonia	No.
Progressive infiltrate + ≥ 2 clinical criteria	19
Progressive infiltrate + 1 clinical criteria	13
Progressive infiltrate + 0 clinical criteria	2
No progressive infiltrate	4
Total	38

a Clinical criteria defined as white blood cell count ≥ 12 or <4, temperature ≥ 38°C or < 36°C, purulent sputum.

## Discussion

In our analysis, the overall rate of antibiotic escalation in response to a positive sepsis screen was 22.7%, and a new infectious diagnosis was identified in 13.2% of all cases with a positive sepsis screen. Additionally, for cases in which antibiotics were escalated without a new infectious diagnosis, antibiotics were promptly deescalated within 3 days in most cases, and only 3.0% of positive sepsis screen cases experienced prolonged antibiotic exposure without a documented infectious etiology. Our analysis showed that the inpatient sepsis screen led to a substantial number of cases with a new infectious diagnosis, and the risk of prolonged antibiotic exposure, and without a clear infectious source was low. Contrary to concerns, our findings indicate that the inpatient sepsis screen was not a major driver of potentially unnecessary antibiotic utilization and that it may have positively contributed to identifying true infections in hospitalized patients.

Our antibiotic escalation rate of 22.7% was lower than what has previously been described in the literature. A study by Gyang et al^20^ that similarly evaluated a nurse-driven sepsis screen in non-ICU patients with similar positive screen criteria showed an antibiotic escalation rate of 56%. One possible explanation for this discrepancy is the frequency of sepsis screen. Our sepsis screen reviewed vital signs and laboratory results from 12 hours prior to screening compared to 8 hours in the study by Gyang et al. The shorter review period may suggest that the positive screen in the Gyang study represented a more acute hemodynamic change in patients, which then prompted a higher escalation rate in response. Thus, incorporating acuity of presentation or decreasing the review period for the sepsis screen may be a reasonable approach to increase specificity of the screen and decrease alert fatigue, which has been shown to be problematic for many automated sepsis screens.^21^ Further studies are needed to ensure that sensitivity is not compromised and that true infections are not missed when making such changes.

Our study is also the first to evaluate the specific etiology of infection after a positive screen. Previous studies have evaluated diagnostic accuracy of sepsis screens by defining true sepsis based on *International Classification of Disease* codes for sepsis.^14^ The use of administrative data has been shown to be inaccurate, and clinical diagnosis of sepsis is also highly variable and subjective.^22,23^ By identifying specific documented infections on chart review, we provide a deeper insight on the infectious etiologies of sepsis identified by the sepsis screen.

However, the new infection rate in our study must be interpreted with caution. Pneumonia was the most common infectious diagnosis made after antibiotics were escalated, which is not surprising given that pneumonia has been described as the most common healthcare-associated infection in a number of point-prevalence surveys.^24,25^ However, only half of the pneumonia cases in our study met clinical criteria outlined in the IDSA/ATS hospital-acquired and ventilator-associated pneumonia guidelines,^15^ which raises concerns that many of our pneumonia cases may have been diagnosed inappropriately. This observation is in line with previous studies. Various studies have reported overdiagnosis and misdiagnosis of pneumonia, reporting a misdiagnosis rate of up to 58%.^26–28^ Additionally, there is precedent from the CMS community-acquired pneumonia mandate, whereby the mandate, which required initiation of antibiotics within 4 hours of suspected pneumonia, contributed to increased overdiagnosis of pneumonia and unnecessary antibiotic use.^29,30^ Given the number of cases with potentially inappropriate diagnosis of pneumonia, our true rate of inappropriate antibiotic prescription may be higher. Stewardship efforts to improve clinical diagnostic accuracy of pneumonia are needed.

Not surprisingly, presence of fever and abnormal lactate levels were significant predictors of antibiotic escalation. Abnormal lactate was previously a component of the definition of severe sepsis, and measuring a lactate is a process measure of the SEP-1 mandate.^9,16^ However, the utility of lactate in sepsis care has been called into question as outlined by the IDSA position paper on the SEP-1 mandate, which recommends removal of lactate measurement from SEP-1 due to concerns for indiscriminate testing leading to unnecessary fluid administration, diagnostic evaluations, and antibiotic prescribing.^12^ In our study, it is unclear whether lactate levels led to unnecessary or inappropriate antibiotic escalation. Regardless, as our institution allows for initiation of components of the sepsis bundles, including obtaining a lactate, without physician orders for patients who screen positive for the sepsis screen, there is theoretical concern for indiscriminate lactate testing, which may have influenced physicians to prescribe unnecessary antibiotics. Additional studies are needed to better characterize the risk of lactate testing on inappropriate antibiotic prescription to better inform future diagnostic stewardship efforts.

Our study had several limitations. First, the retrospective nature of the study limited our ability to fully capture the clinical picture of each case. Although we thoroughly reviewed daily progress notes to identify new infectious diagnoses and alternative causes of the positive sepsis screen, that assessment was limited to the chart documentation. Additionally, we only abstracted the most abnormal labs and vitals that were recorded anytime during the review period, as opposed to abstracting the most recent vital signs from time of antibiotic escalation; thus, the hemodynamic profile reflected by our abstracted vital signs may not have been consistent with the hemodynamic profile at the time of antibiotic escalation. Lastly, our sample was limited to 1 healthcare system. The 2 hospitals included in our study are academic medical centers with many patients with complex medical comorbidities, and our findings may not be directly generalizable to other healthcare facilities with lower-acuity patients.

In summary, in our cohort of patients, a positive inpatient sepsis screen led to the patient being diagnosed with a new infection in 13.2% of cases. Additionally, antibiotics were promptly deescalated within 3 days in most cases when antibiotics were escalated without a clear infection, and only 3.0% of cases experienced prolonged antibiotic exposure without a documented infectious source. Antibiotic stewardship and sepsis treatment often have conflicting interests; however, as suggested by our study, the push for early recognition and treatment of sepsis via routine sepsis screen largely did not seem to interfere with judicious antibiotic use. Our results must be interpreted with caution because our study also indicated that inappropriate diagnosis of pneumonia may be driving antibiotic use for patients with positive sepsis screen. Furthermore, we found that abnormal lactate was a significant predictor of antibiotic escalation, which may suggest an opportunity for future studies to inform diagnostic stewardship efforts. Because management of sepsis and drug-resistant organisms remain national healthcare priorities, further studies are needed to establish the most optimal treatment approach for sepsis while minimizing the risk of unnecessary antibiotic use.

## Supplementary material

For supplementary material accompanying this paper visit https://doi.org/10.1017/ash.2021.232. click here to view supplementary material
