# Supplementary material for: Evaluation of antibiotic escalation in response to nurse-driven inpatient sepsis screen
Source: Antimicrob Steward Healthc Epidemiol. 2021 Dec 3;1(1):e59. doi: 10.1017/ash.2021.232 (PMC9495422; doi:10.1017/ash.2021.232)
Supplement: Supplementary file 1 [file S2732494X21002321sup001.docx]

**Supplementary materials**

Spplementary Table 1: Questions and criteria evaluated on the nurse-driven sepsis screen

| Questions/criteria | Answers |
| --- | --- |
| 1. Do you suspect this patient has a new or worsening infection? | Yes/No |
| 1. In last 12 hours, temperature <36C or >38.3 | Yes/No |
| 1. In last 12 hours, HR >90 beats per minute | Yes/No |
| 1. In last 12 hours, RR > 20 breaths per minute | Yes/No |
| 1. In last 12 hours WBC >12 or <4 or Bands >10% | Yes/No |
| 1. Total number of positive SIRS criteria | Total # of “Yes” to above criteria |
| 1. In last 12 hours, SBP<90 or MAP<65 | Yes/No |
| 1. In last 12 hours creatinine >2 | Yes/No |
| 1. In last 12 hours bilirubin >2 | Yes/No |
| 1. In last 12 hours platelet count <100,000 | Yes/No |
| 1. In last 12 hours INR>1.5 or aPTT>60 | Yes/No |
| 1. In last 12 hours lactate level >18 | Yes/No |
| 1. In last 12 hours respiratory dysfunction | Yes/No |
| 1. In last 12 hours altered mental status | Yes/No |
| 1. Patient has suspected organ dysfunction not related to chronic condition to medication | Yes/No |
| 1. Is the patient positive for severe sepsis/septic shock | Yes/No |

*questions 2-13 are auto-populated in the electronic medical record

Supplementary Table 2a: Ranking of antibiotics based on spectrum of activity against Gram negative bacteria

| Rank | Antibiotics |
| --- | --- |
| 1 | ceftazidime-avibactam, ceftolozane-tazobactam |
| *2* | meropenem, imipenem |
| 3 | ertapenem, cefepime, ceftazidime, piperacillin-tazobactam, aztreonam, aminoglycosides |
| 4 | quinolone, third generation cephalosporin |
| 5 | aminopenicillin, second generation cephalosporin |
| 6 | penicillin, first generation cephalosporin |

Supplementary Table 2b: Ranking of antibiotics based on spectrum of activity against Gram positive bacteria

| Rank | Antibiotics |
| --- | --- |
| 1 | linezolid, daptomycin |
| 2 | vancomycin, ceftaroline |
| 3 | doxycycline, clindamycin, trimethoprim/sulfamethoxazole |
| 4 | penicillin, aminopenicillin, first generation cephalosporin |
